# Membrane Models and the Pursuit of Coherence

**DOI:** 10.1021/acs.biochem.6c00367

**Published:** 2026-07-22

**Authors:** Troy A. Kervin, Ziyao Zhang, Matthew Clark, Michael Overduin, Peijun Zhang

**Affiliations:** † Division of Structural Biology, Nuffield Department of Medicine, University of Oxford, Oxford OX3 7BN, U.K.; ‡ Department of Engineering Science, University of Oxford, Parks Road, Oxford OX1 3PJ, U.K.; § Department of Biochemistry, Faculty of Medicine and Dentistry, 3158 University of Alberta, Edmonton T6G 2H7, Canada; ∥ Diamond Light Source, Harwell Science and Innovation Campus, Didcot OX11 0DE, U.K.; ⊥ Chinese Academy of Medical Sciences Oxford Institute, University of Oxford, Oxford OX3 7DQ, U.K.

## Abstract

We reexamine major membrane models against current evidence. Each addresses important questions, but not all are consistent with the available data. Seeking coherence, we propose the *strong proteolipid code*, a framework of versioned submodels building on an earlier proposal that clarified the relationship between protein and lipid distributions. The first of these submodels introduces glue-field duality as a structure for lipid-mediated protein interactions, classifies protein islands, and bounds autonomous lipid behavior.

## Graphical abstract

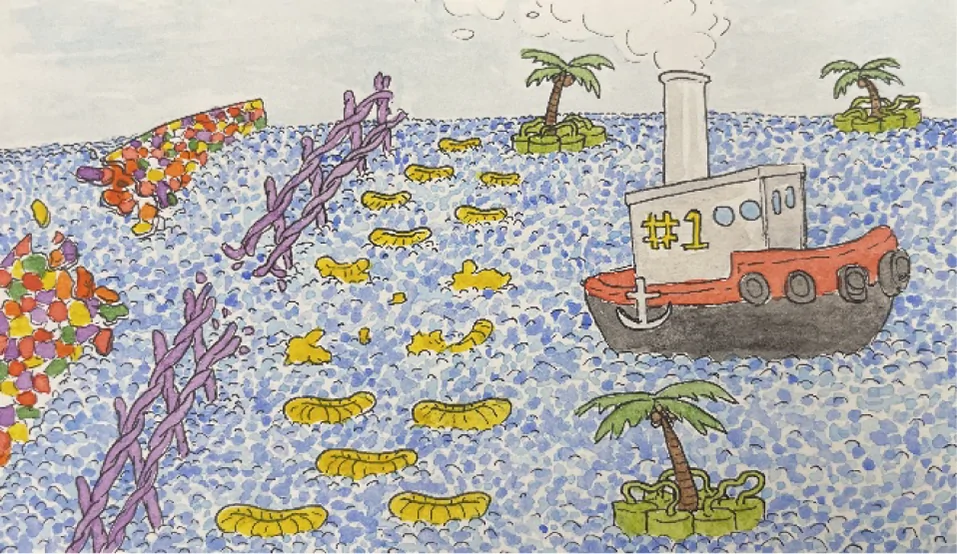

## Introduction

Membrane models have a history of productive succession. A century ago, the lipid bilayer was considered a complete description.^1^ Some decades later, proteins were added to the bilayer, with several different arrangements proposed,^2–4^ culminating in the fluid mosaic model, which became what many considered a consensus for a reasonably long time.^5^ The fluid mosaic model and its successors left many phenomena unexplained,^6^ and foremost among these was membrane zonation: how membrane components organize into uniquely functional subregions.

Many models have since been proposed to account for zonation and other gaps, but the field has become fragmented, with overlapping and often contradictory accounts.^7,8^ This motivates us to reexamine major membrane models against current evidence. We cover the fluid mosaic model,^5^ original lipid raft theory,^9^ lipid self-complexes,^10–12^ Levental and Veatch’s phase separation ideas,^13–15^ Kusumi’s picket-and-fence and tiered mesoscale domain models,^16,17^ competing bilayer asymmetry models,^18–20^ protein island variability,^21,22^ boundary lipids and fingerprints,^23,24^ lipid-mediated protein interactions,^25–27^ lipid recognition for subcellular localization,^28^ and the proteolipid code.^29^

The idea of a “membrane code” or “lipid code” has gained traction in recent years, primarily within phosphoinositide signaling,^29–33^ but declaring that a code exists does not supply a testable mechanism. The proteolipid code specifies a main mechanism relating protein and lipid distributions, but its simplicity limits what it can predict. We therefore offer the *strong proteolipid code*, following the philosophical and scientific convention where the strong form of a claim commits to more and is therefore easier to refute, while the weak form is more permissive. To avoid the field’s habit of revising models indefinitely rather than retiring them, the strong proteolipid code should be thought of as a series of versioned submodels. We assemble Version 1, the first of these submodels, drawing on the analysis that follows.

## Membrane Models

### Fluid Mosaic Model

The fluid mosaic model unified decades of work on membrane structure by proposing that proteins diffuse freely in a lipid bilayer solvent, yielding a random distribution under unperturbed conditions.^1,5^ Its central insight was perhaps to replace static pictures of membrane architecture with a dynamic one. Real membranes, however, typically violate the model’s assumptions in numerous ways. Many zones are not fluid: crystalline or quasi-crystalline lattices are common.^34–38^ Many zones are not mosaic: these lattices are periodic. Many zones are not bilayers: lipids adopt nonbilayer configurations including isotropic formations and inverted hexagonal states, particularly during membrane fusion and fission and in thylakoid and mitochondrial inner membranes.^39–45^ And proteins are not randomly distributed, as the rest of this article will elaborate. Attempts to modify the fluid mosaic model have been made,^8,14^ but none satisfactorily explain membrane zonation. We propose salvaging it as the “Singer–Nicolson regime,” where proteins are weakly interacting and the lipids form a bilayer.

### Original Lipid Raft Theory

In 1997, Simons and Ikonen proposed the lipid raft theory: a bold claim about cholesterol–sphingolipid self-clustering into platforms and subsequent protein recruitment to these platforms^9^ (Figure 1). Their proposal addressed the zonation gap in the fluid mosaic model with a concrete, mechanistic account of membrane sorting and stimulated an enormous body of experimental work. However, their wording, syntax, and argument structure conveyed a sequential mechanism where lipids organize first and proteins follow, as did prior work by Simons.^46,47^ We hereafter refer to this as “lipids-first.” Opposition to the lipids-first mechanism was articulated early. Edidin stated, “proteins recruit lipids, rather than the opposite”,^48^ most likely meaning that lipids do not form *platforms* that recruit proteins, since a protein binding a lipid is equivalent to a lipid binding a protein, while recruitment to a lipid platform implies the platform’s prior assembly. Later critiques used the word “preexisting” to refer to such platforms.^49,50^ Immunogold electron microscopy showed that the spatial distribution of putative raft proteins is incompatible with the lipids-first mechanism,^51^ and evidence for autonomous lipid clustering under physiological conditions has been limited, as reviewed below. In response to counterevidence, the lipid raft theory underwent many modifications,^15,17,52–56^ causing confusion in the literature,^7,57,58^ though cognizant authors have drawn attention to this.^59–62^ Philosophers of science argue against this type of endless redefinition.^63,64^

**Figure 1:**
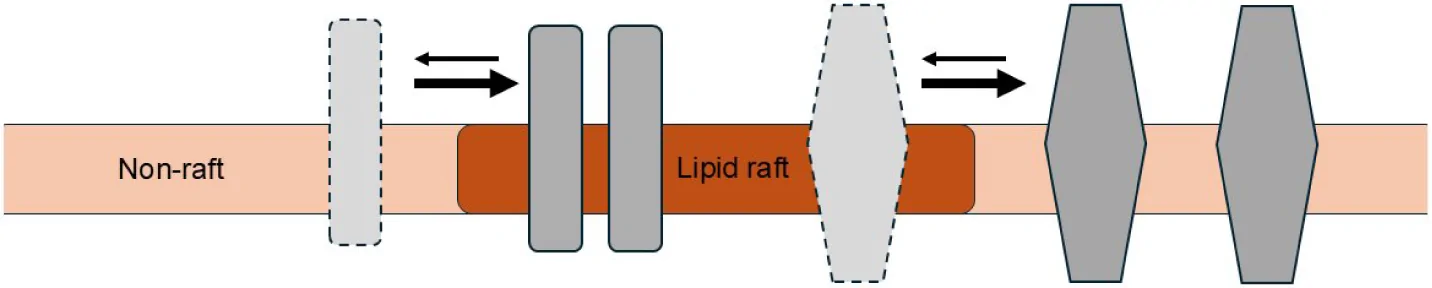
Lipids-first mechanism. Proteins (gray shapes) are partitioned into preassembling lipid raft or nonraft regions based on their preference for one or the other. This schematic is consistent with representations used by lipid raft proponents.^65^

### Lipid Clusters

A natural question arising from the lipid-raft theory is whether lipids autonomously form large clusters, which is necessary but not sufficient for the lipids-first mechanism. Evidence for lipid self-clustering in physiological environments is nuanced, identity-dependent, and limited. It has been proposed that nearly all membrane cholesterol exists in stoichiometric complexes with phospholipids based on extraction kinetics and monolayer studies at an air–water interface.^10–12,19,66,67^ This model is attractive in its simplicity and makes quantitative predictions about cholesterol availability. However, neither coarse-grained nor atomistic MD simulations at physiological temperatures have produced stoichiometric cholesterol–phospholipid complexes or cholesterol–sphingolipid platforms,^68–70^ and cryo-EM structures consistently resolve cholesterol at protein-specific binding sites rather than as phospholipid–cholesterol units.^35,71–74^ On the other hand, cholesterol dimers, tetramers, and perhaps even higher oligomers are demonstrated by solid-state NMR and MD simulations^75^ and observed in many crystal structures and some cryo-EM structures,^71,73,76–79^ although some are likely artifacts.^80^ Phosphoinositide and phosphatidylserine clustering may be mediated by divalent cations, and gangliosides may cluster through sialic acid hydrogen bonding, but these clusters are small and involve specific chemistries and ionic conditions.^81–87^ We believe that autonomous lipid clustering may be common for some species but rare for most, and when it does occur, clusters are typically small.

### Phase Separation Models

Canonical phase separation is an equilibrium framework.^88–90^ When used to describe nonequilibrium phenomena, it is often necessary to analyze the context in which the term “phase separation” is used to extract the intended meaning. Levental et al. argue that membranes are divided into merely two phases, by which they appear to mean states resembling equilibrium phases.^13,91^ This derives from decreasing the temperature of artificial and derived systems, where something akin to two-phase spinodal decomposition is indeed observed.^91,92^ However, this extrapolation commits the fallacy of composition: simplified systems are used precisely because this phenomenon is not observed in more complex, physiological membranes; hence, the whole is assumed to have the properties of its isolated parts.^93^ The proposal that lipid distributions around membrane protein clusters are “phases” that interpolate between binary, artificially induced ones descends from this idea^14,15,93^ and was justified by assuming causation from correlation.^93^ As explained below, lipid distributions around proteins are expected to be heterogeneous and protein-specific,^24^ not interpolations between two bulk phases. Hereafter, we resist the phase separation framework to avoid ambiguous epistemic commitments that conflate equilibrium and nonequilibrium physics.

### Actin Fence Models

Anomalous diffusion patterns revealed by single-particle tracking inspired the picket-and-fence model, wherein integral proteins anchored to cortical actin filaments corral membranes into compartments.^16,94,95^ This drew productive attention to cytoskeletal contributions to membrane structure. However, compartmentalized diffusion occurs in systems lacking a cytoskeleton.^96–98^ Actin-disrupting drugs enlarge compartment sizes but fail to abolish compartmentalized diffusion,^94,99^ and changing total membrane protein content changes diffusion rates independently of actin or spectrin.^100,101^ The model’s central defense, that diffusion in isolated membranes stripped of actin recovers artificial-membrane speeds, is undermined by the finding that chemical isolation depletes membrane protein content, so faster diffusion in isolated systems reflects a simpler environment with reduced crowding.^101^ Simulations also show anomalous compartmentalized diffusion without a cytoskeleton.^102,103^ Anomalous protein and lipid diffusion appears to result from the complex proteolipid environment rather than from a single protein fence.^100^ The picket-and-fence model was later elaborated into a tiered architecture in which “lipid rafts” sit nested within the actin-bounded compartments,^17^ a hybrid that inherits the limitations of both parent models.

### Bilayer Asymmetry

The canonical model of phospholipid asymmetry places phosphatidylserine, phosphatidylethanolamine, and phosphoinositides in the cytoplasmic leaflet and sphingomyelin and phosphatidylcholine in the exoplasmic, actively maintained by flippases, floppases, and scramblases.^104–107^ This pattern is routinely disrupted: phosphatidylserine is commonly externalized,^108–111^ phosphatidylethanolamine appears on the outer leaflet during cytokinesis,^112^ organelle membranes maintain entirely different profiles,^113,114^ and even the phosphatidylcholine distribution depends on acyl chain saturation rather than the headgroup alone.^107^ Moreover, the distribution of cholesterol remains one of the most contested questions in membrane biophysics. Reported values for outer leaflet cholesterol enrichment range from 12–13% in mouse synaptic membranes to 57–72% depending on buffer conditions in human erythrocytes.^115–118^ Doktorova et al. propose approximately 3-fold exoplasmic cholesterol enrichment driven by a phospholipid abundance imbalance,^18^ while Steck and Lange predict 2:1 enrichment through their stoichiometric cholesterol–phospholipid complex model.^19^ While these offer quantitative, predictive accounts, the measurements on which they rely face challenges. Cholesterol sensors have been questioned as unreliable across different lipid compositions,^119^ and it has been pointed out that if the great majority of cholesterol were free in the outer leaflet, probes should readily detect it.^20^ We argue that this debate is ill-posed at the whole-membrane level. Different zones maintain different lipid compositions, and global measurements necessarily average over them. The wide range in reported values is precisely what one would expect if zone-level or more broadly cell- and organism-level asymmetries vary substantially. The problem is compounded by the inevitability that major methods perturb the distribution they seek to measure. This underscores the need to frame questions within the context of zonation.

### Protein Island Variability

It is well-established that membrane proteins cluster into islands of varied size, shape, and stability,^120^ but the organizational principles governing these distributions remain unresolved. Many protein islands follow a size distribution where the probability of observing an *n*-mer decreases with *n*.^21,22,121^ A subset of these clusters has been termed higher-order transient structures (HOTS), reflecting interactions that are specific but transient.^22^ This monotonically decreasing pattern can be observed even in the absence of attractive interactions at sufficiently high concentrations (Figure 2A). What could break this? Geometric closure produces obligate oligomers,^122^ cooperative growth produces monomers coexisting with completed shells,^123^ competing attraction and elastic repulsion select finite cluster sizes,^124,125^ and active processes can maintain a fixed clustered fraction regardless of the expression level.^126^ Elongated or branched cluster morphologies are also common, arising from anisotropic interactions, ramified aggregation at high density (Figure 2C), or other mechanisms.^127–130^ Increasing or decreasing protein concentration can also affect the shape of the distribution.^22^ Categorization of protein islands would be valuable in a membrane model.

**Figure 2:**
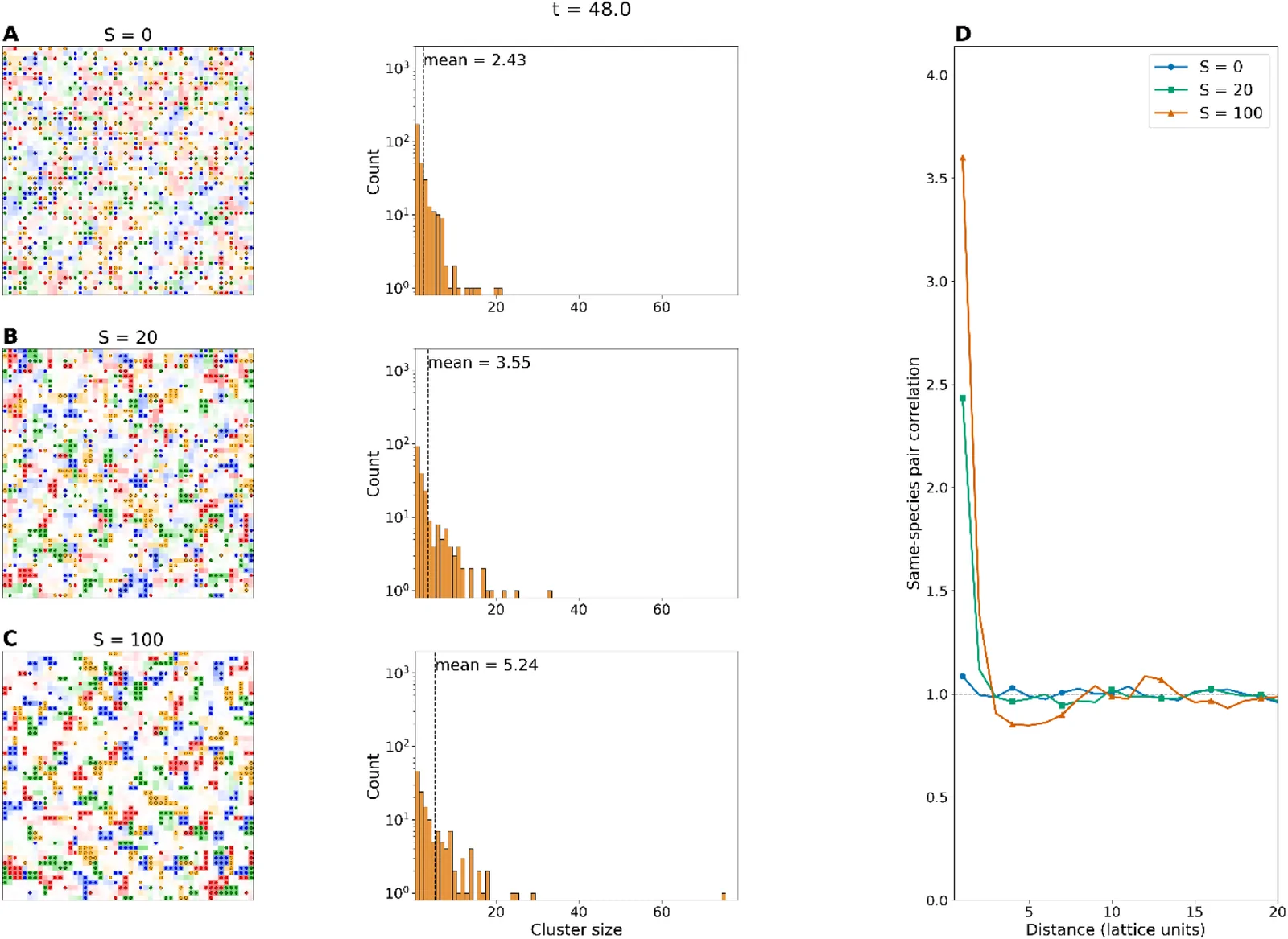
Lattice model of membrane protein clustering at three adhesion strengths on a 50 × 50 grid, largely following a self-assembly model of higher-order transient structures (HOTS).^22^ Four protein species (colored dots) diffuse at fixed numbers; a protein’s probability of staying put increases with its number of same-species neighbors, set by a stickiness parameter *S*. Each species imposes a characteristic lipid preference on its occupied site which spreads into a surrounding patch. Background color indicates the locally dominant lipid species, with saturation proportional to dominance (white means well-mixed). Unoccupied sites relax toward uniform composition, and the lipid field diffuses with stochastic noise.^131^ Rows show *S* = 0 (A), 20 (B), and 100 (C). Adjacent to each grid, a histogram gives the distribution of protein cluster sizes (all species pooled) with the mean marked with a dashed line. The lipid field does not feedback on protein diffusion in this minimal model. (D) Same-species pair correlation for each regime (averaged over the four species) versus separation distance in lattice units: correlation = 1 is the random baseline. The animated version and source code are available in the Supporting Information section.

### Boundary Lipids and Fingerprints

The concept of boundary lipid layers around membrane proteins was introduced in the 1970s.^23^ This was an important step in recognizing the role of proteins in shaping membranes. However, boundary lipids were typically treated as relatively uniform.^132,133^ Similarly, it was proposed that cholesterol–phospholipid complexes discussed above could assemble around proteins into long-lived shells.^49^ The lipid fingerprint concept challenged this picture,^24^ specifying that each membrane protein has a unique, heterogeneous lipid distribution shaped by its structure and the surrounding lipid environment (Figure 3). This is strongly supported by molecular dynamics simulations and cryo-EM structures, which show each protein’s unique and multivalent lipid interactions.^81,134^ These distributions include lipid species enrichment as well as curvature, ordering, and other deformations. The fingerprint concept excludes one-to-many descriptions in which a single type of shell or compositional state applies to diverse proteins, including the recent claim that protein clusters are surrounded by states interpolating between artificially induced binary equilibrium phases.^15,93^ Instead, it underscores that the membrane is a giant combinatorial code in which each protein contributes a unique signature.

**Figure 3:**
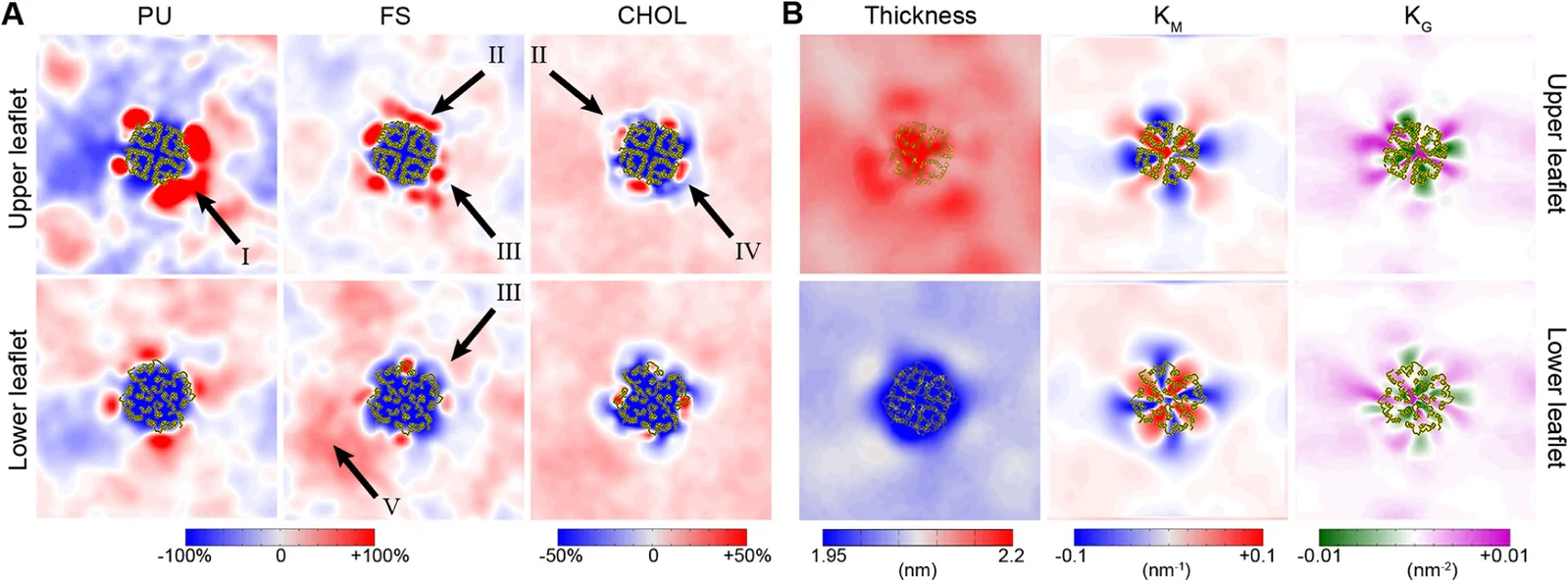
Lipid fingerprint for a membrane protein.^24^ (A) Heterogeneous lipid density with polyunsaturated (PU) lipids, fully saturated (FS) lipids, and cholesterol shown. Important features are labeled I–V. (B) Thickness, mean curvature, and Gaussian curvature in the fingerprint. Reproduced from Ref. 24. https://doi.org/10.1021/acscentsci.8b00143. Copyright 2018 American Chemical Society.

### Lipid-Mediated Protein Interactions

When multiple proteins inhabit the same bilayer, the lipid perturbations they create, which we may now understand as fingerprints,^24^ may overlap and generate effective interactions from a distance.^25–27^ This body of theoretical work typically models these interactions as decaying with an inverse power of distance.^25,135^ Two important signatures are curvature and ordering; similar curvatures or ordering arrangements attract, while dissimilar ones repel.^25,135^ At a shorter range, discrete lipids can mediate bridge-like interactions between proteins, termed lipid glue^35,136–138^ (Figure 4). These two regimes have largely been studied by separate communities: continuum theorists treating the long-range case and structural biologists resolving the glue. Yet, they appear to represent limiting behaviors of a single continuum: field-like interactions transmitted through numerous lipid self-interactions at long-range, and glue-like interactions mediated by few lipids at short-range. We return to this duality below.

**Figure 4:**
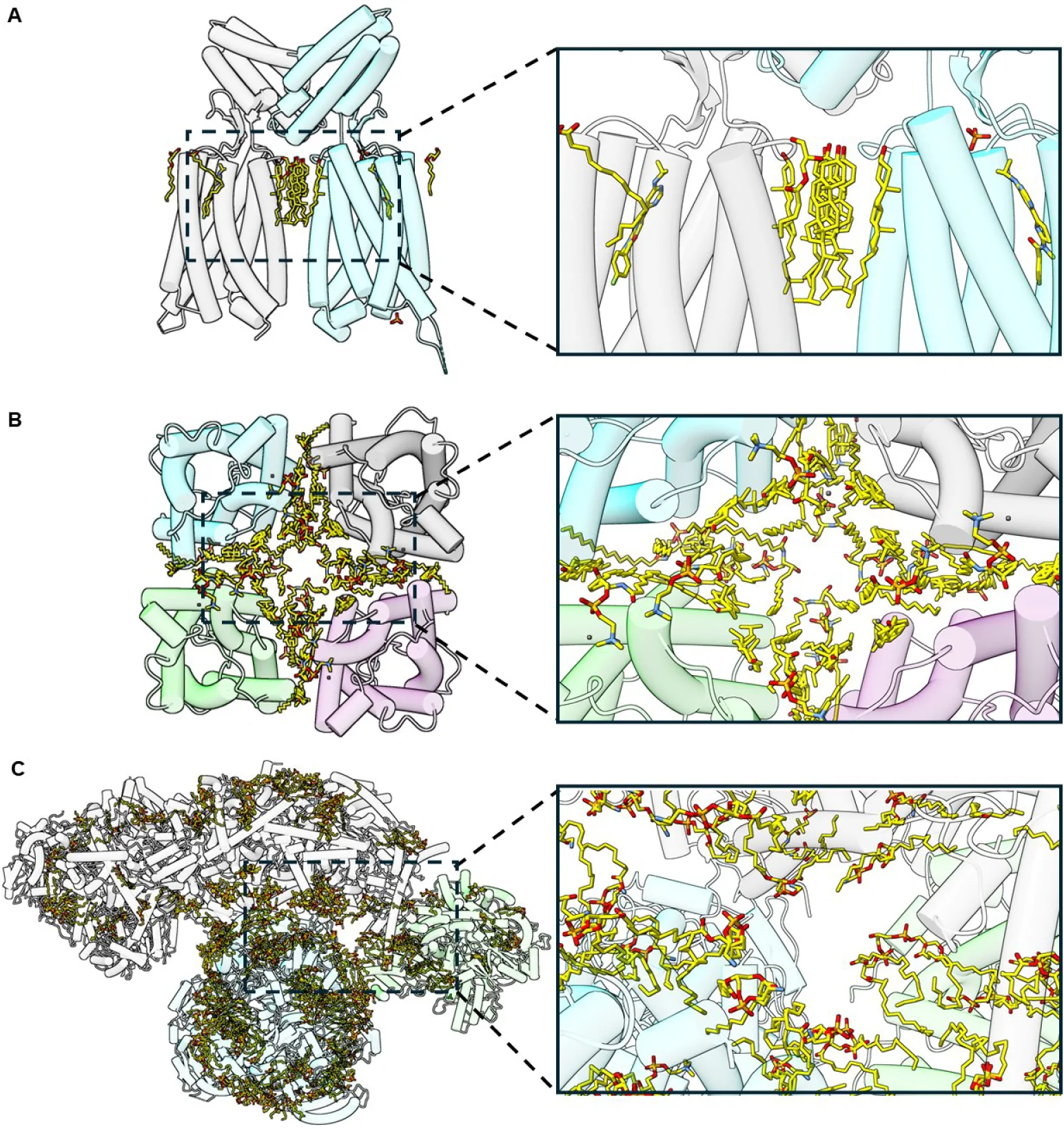
Lipid glue. Lipids in stick representation are shown stabilizing protein complexes represented as cartoons with tube helices and with protomers colored light gray, cyan, lime, or purple. Stick atoms are colored yellow for carbon, orange for phosphorus, red for oxygen, cornflower blue for nitrogen, and green for fluorine. (A) Crystal structure of the G protein-coupled metabotropic glutamate receptor 1 with cholesterol glue (PDB ID: 4OR2).^73^ (B) Crystal structure of aquaporin-0 with cholesterol and sphingomyelin glue (PDB ID: 8SJY).^35^ (C) Cryo-electron microscopy structure of a mitochondrial respiratory supercomplex with glue of various lipids including phosphatidylethanolamine, phosphatidylglycerol, phosphatidylcholine, and cardiolipin glue (PDB ID: 8UGH).^81^ Created with UCSF ChimeraX.

### Lipid Recognition by Peripheral Proteins

The ability of proteins to recognize sets of lipids to localize their function, which have been called “lipid codons” or “lipidons,” was popularized in the 2000s under the name coincidence detection.^28,139,140^ A growing body of lipid-bound protein structures, molecular dynamics simulations, and biochemical assays supports the lipidon concept, extending it from organelle to suborganelle resolution.^32,141–143^ Lipidon recognition should now be understood as a remodeling phenomenon often involving tens or hundreds of distinct molecules that are locally rearranged.^143^ This is central to endocytosis,^144^ exocytosis,^145^ membrane fusion and fission,^146^ lipid trafficking,^147,148^ signal transduction,^30^ and pathogen lifecycles.^149,150^ Important signals that allow lipidons to be distinguished from each other include electrostatic attraction to charged lipid headgroups, hydrophobic insertion into the membrane interior, stereospecific recognition, and coincidence detection of protein motifs or ions.^28^ It is believed that the electrostatic signal provides an initial long-range attraction that brings the protein to the membrane surface while hydrophobic and stereospecific interactions add specificity.^151^ In eukaryotes, at least one of the eight phosphoinositides (counting the parent molecule) is usually detected in lipidons, with readers found in at least 70 distinct protein folds across human, microbial, and viral proteomes.^139^ Overall, lipidons explain how a large variety of peripheral proteins find their subcellular destinations and execute their functions. However, the lipidon concept does not explain how the lipidons themselves arrive at the right locations, a question that partially motivated the proteolipid code.^29^

### Proteolipid Code

The proteolipid code was proposed as a general model for membrane structure.^29^ It combined several theories and philosophical principles, including how membranes participate in the flow of biological information. Its two main theories are rejection of the lipids-first mechanism and adoption of lipid fingerprints. Taken together, these two theories imply that rather than being recruited to lipid-only submembrane platforms, proteins cluster together with their lipid fingerprints to form protein islands with a collective fingerprint.^24,29,152^ It also follows that protein-free regions (void zones), which are left behind when proteins cluster, have a different lipid composition from protein-containing zones.^29,153^ This is the proteolipid code’s main mechanism. The lipidon concept connects naturally as it describes how peripheral proteins participate in or recognize the zones supplied by this fundamental principle. Lipidons are lipids within any zone whose identity encodes subcellular location, and they become fingerprint lipids of a given protein once recognized by that protein. Several theories discussed above fall outside the proteolipid code by endorsing the lipids-first mechanism, rejecting lipid fingerprints, or both.^9,14,15,49^ The model can also be represented with sheaf theory, formalizing how molecules cohere across zones into a globally consistent organization.^154^ Overall, the proteolipid code provides a sturdy but deliberately sparse foundation on which more detailed models can be built.

### Strong Proteolipid Code

Here we propose the *strong proteolipid code*: a family of submodels whose weak form is the original proteolipid code. Version 1, the first implementation of this framework, aims to unify phenomena that have traditionally been treated as separate, thereby providing a more coherent account of their underlying relationships. It augments the core mechanism by incorporating molecular and statistical detail, proposes that autonomous lipid behavior is considerably more limited than is often assumed in the literature, and provides a more structured framework for protein–lipid coupling. Several of its predictions are intentionally formulated to distinguish it from competing models. Version 1 is intended to apply to physiological membranes and may not necessarily extend to *in vitro* or *in silico* systems.

We label each hypothesis [version].[number], so 1.1 denotes the first hypothesis of Version 1.

### Glue-Field Duality

Let us distinguish two limiting behaviors on a continuum:

- Define glue-like behavior as fingerprint-mediated interactions involving few lipids trapped between proteins.
- Define field-like behavior as fingerprint-mediated interactions involving numerous lipids not trapped between proteins.

Glue-like and field-like behavior can coexist, such as when two proteins are close and have glue-like lipids inserted between them while the surrounding lipids drive field-like depletion attraction.^155,156^ “Field lipid” and “glue lipid” can be used as shorthand for the dominant mode of interaction a lipid mediates in each context. We propose a foundational distinction:

**1.1**. Fingerprint-mediated interactions are more sensitive to the perturbation of an individual glue lipid than of an individual field lipid.

This implies that as proteins approach, their interstitial lipid-mediated interaction shifts from relatively smooth to granular, allowing a selection of lipids to be more consequential for protein clustering. We designate this as a central hypothesis mostly because it is broadly applicable (it builds on the main mechanism), helping Version 1 maintain a core structure. This hypothesis is inspired by wave-particle duality as well as gravitational and strong nuclear forces.

### Typology of Protein Islands (1.2)

Membrane protein islands are clusters of membrane proteins, including protein complexes and transient structures.^120^

**1.2.** We hypothesize that membrane protein islands of a given type are distributed in size, by protein count, according to one of the following.

1. Monotonically decreasing with thin tail: higher-order oligomers are progressively rarer, including exponential.^22,157^

2. Monotonically decreasing with heavy tail: decay slower than exponential, including power-law, stretched exponential, and logarithmic series.^22,158,159^

3. Narrow-peaked: peaked at a specific size above monomer with low variance, including Poisson with sufficiently high mean and the monodisperse (delta function) limit.^125,160,161^

4. Right-skewed peaked: peaked at a preferred size above monomer with a long tail toward larger islands, including log-normal.^162–164^

5. Bimodal or multimodal: coexistence of two or more distinct populations at preferred sizes, including periodically spaced peaks.^159,165–167^

This typology applies to one defined class of protein islands, not to aggregate distributions. Its value lies in what it excludes, such as monotonically increasing, uniformly flat, and strongly left-handed distributions. Observation of any such distribution would force a revision.

### Additional Predictions

In the hypotheses below, the lipid fingerprint is the protein-perturbed lipid environment that is distinguishable from the baseline.^24^

*Fingerprint and Void Structure*

**1.3**. Mean fingerprint lipid residence time decreases monotonically with distance from the protein on average across fingerprints.^152,168^

**1.4**. Higher and lower mean lipid acyl chain orders relative to void zones both occur commonly across fingerprints.

**1.5**. Lipid fingerprints are more often enriched in negatively charged lipids relative to void zones.^169^

*Protein Islands*

**1.6**. The importance of direct protein–protein interactions is negatively correlated with that of lipid glue for stabilizing membrane protein complexes.^170^

**1.7.** Glue antagonists disassemble protein complexes by outcompeting potential lipid glue. The extent of disassembly increases with the antagonist’s concentration.^171^

*Lipid–Lipid Interactions*

**1.8**. Lipid complexes of ten or more molecules do not form autonomously; those that exist require proteins for stability.^81^

**1.9**. Cholesterol dimers are the most common lipid complexes in mammalian plasma membranes.^75^

**1.10**. Cholesterol–phospholipid complexes do not occur unless stabilized by proteins. This challenges the cholesterol–phospholipid stoichiometric complex model.^11^

**1.11**. Cholesterol has stronger interactions with saturated aliphatic chains than with unsaturated chains.^172–175^

*Lipid Bilayer Structure*

**1.12**. Nonbilayer lipid configurations occur more frequently in lipid fingerprints than in void zones.^24^

**1.13**. Membrane fusion and fission always proceed through nonbilayer intermediates.^146,176^

**1.14**. Lipid leaflet asymmetry deviates further from the bilayer-wide average in lipid fingerprints than in void zones.

**1.15**. Cholesterol leaflet asymmetry varies dramatically between zones, cell types, and organisms. Neither cytoplasmic nor extracellular leaflet enrichment is universal across cell types.^115–118^ This challenges recent models of cholesterol bilayer asymmetry.^18,19^

*Miscellaneous*

**1.16**. Anomalous compartmentalized diffusion is a general feature of complex proteolipid membranes.^100^ Cytoskeletal filaments contribute to this but are not the sole or primary determinant of it, contradicting the picket-and-fence model.^16^

**1.17**. Lipidon recognition by peripheral proteins that are not anchored to the membrane through covalent lipid modifications involves at least one directly bound anionic lipid in most recognition events in eukaryotes.

## Conclusions

Without a holistic framework, models appear to develop around specific bodies of evidence that contradict the broader literature. Several influential proposals face such tensions: the suggestion that nearly all plasma membrane cholesterol forms stoichiometric complexes with phospholipids,^10,11,66^ the division of membranes into two “phases”,^13^ and the attribution of compartmentalized diffusion to a single protein fence.^16^ Models built on these foundations inherit their uncertainties. Cholesterol bilayer asymmetry has been explained almost entirely through cholesterol–phospholipid complexation,^19^ protein clusters have been proposed to induce “phases” interpolating between values from synthetic systems,^15^ and the hypothesized fence has been invoked to limit the growth of “rafts”.^17^

The proteolipid code offers a synoptic model but lacks detail.^29^ Here, we build on it by proposing Version 1 of the strong proteolipid code, a collection of hypotheses representing a current account of membrane structure. This versioning scheme avoids the problem of endless redefinition that degraded the lipid raft theory and allows models to be retired cleanly, their failures documented, and a successor proposed without the field having to relitigate its foundations.

## Supplementary Material





## Data Availability

Source code for Figure 2.

## Notes

Regimes animation (MOV)
Animated version and source code of regimes (ZIP)
